# Development of Postural Stability Index to Distinguish Different Stability States

**DOI:** 10.3390/e21030314

**Published:** 2019-03-22

**Authors:** Nurul Retno Nurwulan, Bernard C. Jiang, Vera Novak

**Affiliations:** 1 Department of Industrial Management, National Taiwan University of Science and Technology, 43 Keelung Road Section 4, Daan District, Taipei City 10607, Taiwan; 2 Department of Neurology, Beth Israel Deaconess Medical Center, Harvard Medical School, Boston, MA 02215, USA

**Keywords:** postural stability index, stability states, ensemble empirical mode decomposition, gait

## Abstract

A key factor for fall prevention involves understanding the pathophysiology of stability. This study proposes the postural stability index (PSI), which is a novel measure to quantify different stability states on healthy subjects. The results of the x-, y-, and z-axes of the acceleration signals were analyzed from 10 healthy young adults and 10 healthy older adults under three conditions as follows: Normal walking, walking with obstacles, and fall-like motions. The ensemble empirical mode decomposition (EEMD) was used to reconstruct the acceleration signal data. Wearable accelerometers were located on the ankles and knees of the subjects. The PSI indicated a decreasing trend of its values from normal walking to the fall-like motions. Free-walking data were used to determine the stability based on the PSI. The segmented free-walking data indicated changes in the stability states that suggested that the PSI is potentially helpful in quantifying gait stability.

## 1. Introduction

Each year, approximately one third of older adults aged >65 years experience falls [1]. Falls can cause physical injuries that may lower the quality of life and health or even lead to death in older adults. Additionally, falls are a common cause of psychological stress and extended hospitalization for older adults [2]. Falls are potentially related to the difficulty in maintaining walking stability. Therefore, quantifying walking stability is potentially key to preventing falls. 

Previous studies focused on the fall detection method [3,4,5]. However, the indexes can only be used to count the number of falls. They detect falls based on the sudden changes in a series of data. Despite the ability to detect falls, the aforementioned methods merely count the number of falls that occur in a given time. Although falls are caused by poor postural stability, it is difficult to determine the stability of the movement if the fall does not occur.

A previous study used dynamic stability to determine postural stability [6]. The acceleration root mean square (RMS), step and stride regularity, and sample entropy (SampEn) are parameters used to measure dynamic stability. Dynamic stability can be used to identify asymmetrical stability patterns related to ageing and illness [7,8,9]. However, the approach may not be as sensitive in discriminating stability patterns in healthy subjects.

In 2014, Cui et al. [10] used ensemble empirical mode decomposition (EEMD) to construct the step stability index (SSI) to discriminate between the walking patterns of fallers from non-fallers. When compared to fall detection methods, the SSI is a more promising approach to evaluate human postural stability because it evaluates the characteristics of movements irrespective of whether or not subjects fall during the evaluation. The SSI can be used to quantify gait dynamics and discriminate non-fallers from fallers although the real question concerns the extent of the stability of the movement of non-fallers. The evaluation of the postural stability of non-fallers may not be as simple as comparing fallers and non-fallers. EEMD was developed to overcome the empirical mode decomposition (EMD) mode mixing issues [11]. EMD decomposes signal data into a set of zero-mean underlying components called intrinsic mode functions (IMF). The main advantage of EMD is that its algorithm depends only on the signal under analysis. However, performance evaluation of EMD compared to discrete wavelet transform (DWT) and wavelet packet decomposition (WPD) showed that the EMD performed the worst in detecting seizures in electroencephalogram (EEG) signals. Machine learning methods, such as random forest (RF), k-Nearest neighbor (k-NN), artificial neural network (ANN), and support vector machines (SVM), were used to predict the accuracy of the EMD, DWT, and WPD [12].

The current study proposed a measure that can distinguish different stability states in healthy subjects using an accelerometer. This study used EEMD and multiscale entropy (MSE) to develop the measure. The performance of EEMD was also evaluated by comparing its performance with the wavelet transform method. The organization of this paper is prepared as follows. The methods section provides the experiment protocol and a brief description of previous postural stability measures as well as the theoretical background behind EEMD, MSE, and wavelet transform methods. Results are then presented in Section 3 and the discussion is in Section 4. Finally, a conclusion section is presented in Section 5.

## 2. Methods 

### 2.1. Subjects

**Figure 1:**
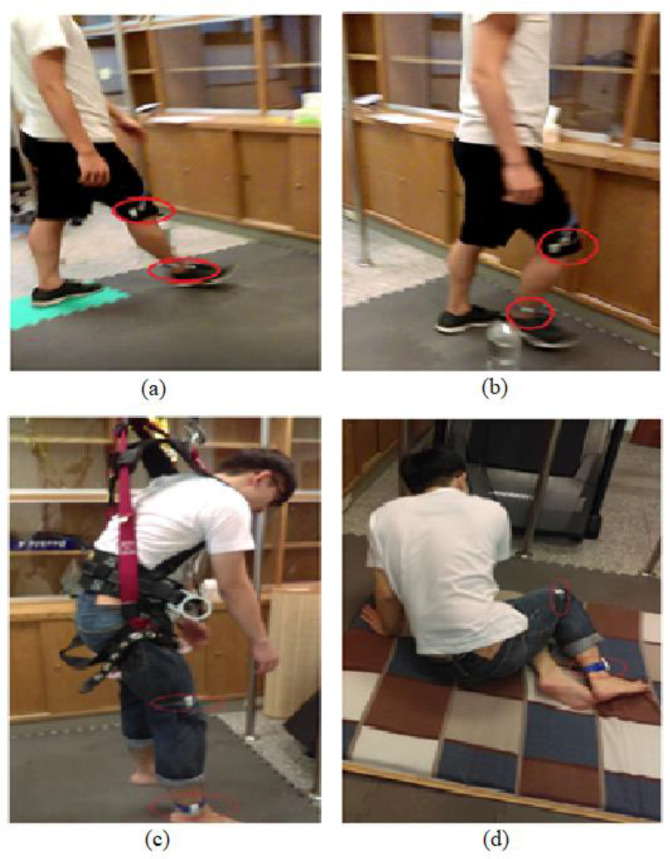
Subject performs the activities: (**a**) normal walking, (**b**) walking with obstacles, (**c**) free-falling, and (**d**) fall-like motions. The older adults did not perform the free-falling task.

Ten young adults (24 ± 0.94 years) and 10 older adults (69 ± 6.77 years) were recruited. All subjects were free of any postural stability-related disorder based on self-reports. The study was approved by the Institutional Review Board and informed consent forms were obtained from all subjects before their participation. The subjects were asked to perform walking, walking with obstacles, free walking, and fall-like motions (Figure 1). Young adults performed free-falling and fall-like motions while older adults only performed fall-like motions. The subjects were instructed to walk at their own pace regardless of the distance for the duration of 60 s. For the fall-like motions, the subjects were asked to sit on a mattress from the standing-still position. The fall-like motion was repeated 10 times. The subjects were allowed to hold on to the pole in front of them while performing the fall-like motions. The results of the pilot study indicated that the subjects did not feel comfortable wearing safety harnesses while performing fall-like motions. The safety harness made the subjects bounce back during the task. For the free-fall task, subjects were asked to jump down from the standing position on the chair to imitate the free-fall. This free-fall task was repeated five times. Wearable accelerometers were attached to the ankle and knee of each subject with the assumption that the ankle and knee corresponded to the most relevant body parts during the experiment. The acceleration data were acquired at 30 Hz and were imported into Matlab R2016a [13] for feature computation.

### 2.2. Subject Characteristics

Twenty healthy subjects with no history of falling in the past two years were grouped by age. Table 1 presents the descriptive statistics of the subjects. The Mann Whitney test was used to compare the two groups. The differences between the two groups were all statistically insignificant, except for age.

### 2.3. Ensemble Empirical Mode Decomposition

Ensemble empirical mode decomposition (EEMD) was introduced to overcome the mode mixing phenomenon in empirical mode decomposition (EMD). Given signal intermittence, mode mixing situations typically occur during the EMD decomposition process. Mode mixing occurs when a single intrinsic mode function (IMF) either consists of oscillations with different frequencies or a signal with a similar frequency is in different IMF components. Mode mixing can change the physical meaning of each IMF component by replacing the part of the IMF and driving it to the next IMF, thereby falsely suggesting that different physical processes may exist in an IMF [11]. 

White noises of the same length were added to the original signal to form a mixture signal, xi(t), and subsequently the EMD decomposition was performed to obtain n layers of IMFs (IMF11, IMF12, …, IMF1n). We assumed that the process was conducted for m times, and this is defined as the number of ensemble members. The original signals were subsequently added by m white noises of the same energies to form m mixture signals. In the current study, white noises of an amplitude that was about 0.2 standard deviation of the signals were added [11]. Each mixture signal, xi(t), was decomposed into n layers of IMF components. Thus, there were m x n IMF components, where m denotes the number of ensemble members and n denotes the nth layer of the IMF. The decomposed results were averaged and subsequently each layer of the IMF was calculated as follows:

(1) $$IMF_{n}=\frac{1}{m}\overset{m}{\underset{i=1}{\sum }}IMF_{n}$$

The purpose of adding the white noise of the finite amplitude to the signal was to populate the whole time-frequency space uniformly with the constituting components of different scales. The white noise cancels each other out in the time-space ensemble mean. Thus, only the true and physically meaningful signal can survive in the EEMD [11].

### 2.4. Wavelet Transform

Wavelet transform decomposes a signal into a set of basic functions by scaling and shifting the mother wavelet function. These basic functions are called wavelets because they wave up and down across the axis, and integrate to zero. Wavelets are highly effective in analyzing non-stationary signals, such as for noise reduction [12]. The discrete wavelet transform (DWT) transforms a discrete time signal to a discrete wavelet representation. It converts an input series, x_0_, x_1_, …x_m_, into one high-pass wavelet coefficient series and one low-pass coefficient series.

Wavelet packet decomposition (WPD) is a continuous wavelet transform. The difference between DWT and WPD is how the scale parameter is discretized. The WPD discretizes the scale more finely than DWT. Thus, it gives a better frequency resolution for the decomposed signal [12]. However, the WPD is less stable for signal reconstruction. Whereas the DWT is able to provide perfect reconstruction of the signal upon inversion, and its coefficients can be used to reproduce an exact signal within numerical precision. In the current study, the number of decomposition levels in DWT was selected to be 6, whereas the daubechies4 (db4) mother wavelet function with 4 levels of decomposition was used for WPD [12].

### 2.5. Multiscale Entropy

Following its introduction by Costa et al. [14], multiscale entropy (MSE) was successfully applied to quantify the complexity of signals in different research fields, such as biomedical [15,16,17,18,19], electroseismic [20], and the vibration of rotary machines [21]. 

MSE proposed a method to measure complexity by constructing a consecutive coarse-grained time series by averaging a successively increasing number of data points in non-overlapping windows as follows:

(2) yj(τ)=1τ∑i=(j−1)τ+1jτxi, 1≤j≤Nτ

where τ denotes the scale factor, and the length of each coarse-grained time series is N/τ. For scale 1, the time series, $\left\{y^{\left(1\right)}\right\}=\left\{x_{1},x_{2},x_{3},\ldots x_{N}\right\}$, simply corresponds to the original time series. The length of each coarse-grained time series is equal to the length of the original time series divided by the scale factor, τ. Subsequently, sample entropy (SampEn) is calculated for each of the coarse-grained time series plotted as a function of the scale factor as follows:

(3) SampEn(m,r,N)=−ln(A/B)

where A denotes the total number of forward matches of a length, m + 1, and B denotes the total number of template matches of a length, m [22]. 

The complexity index (CI) is obtained from the total value of the SampEn as a function of the scale factor as follows:

(4) $$CI=\;\overset{N}{\underset{i=1}{\sum }}SampEn\left(i\right)$$

### 2.6. Step Stability Index

The step stability index (SSI) was proposed to distinguish the walking patterns of fallers from non-fallers [10]. The magnitude of the acceleration signals is decomposed using EEMD with 8-modes of IMFs. Subsequently, the standard deviations of the IMF1 to IMF4 are used to develop the index as follows:

(5) $$SSI=\frac{SD\;IMF4}{SD\;IMF1+SD\;IMF2+SD\;IMF3}$$

where SSI denotes the step stability index, and SD denotes the standard deviation.

The non-fallers exhibited a higher SSI value that indicated a more stable gait pattern because the energy (as measured by the standard deviation) of the component at the step frequency exceeds the energy of higher frequency components that are potentially related to subtle unsteadiness of stepping. 

The SSI is used to quantify gait dynamics and discriminates between subjects with and without a history of falls. However, it is potentially not sufficiently sensitive to distinguish the postural stability of healthy subjects. The difference of the postural stability in non-fallers is potentially not as evident. Thus, the SSI may not be adequate to measure the likelihood of falls or to provide a better understanding as to humans’ maintenance of stability given that it distinguishes non-fallers from fallers without the ability to understand how postural stability corresponds to the movement of non-fallers.

### 2.7. Dynamic Stability

Dynamic stability parameters include the ratio of root mean square (RMS), step and stride regularity, and sample entropy (SampEn) [6]. The ratio of RMS was used to capture the variability as obtained from the ratio of each axis RMS relative to the resultant vector RMS. The step and stride regularity were used to capture the consistency of the gait. Step regularity was obtained from the primary dominant unbiased autocorrelation coefficient while stride regularity is the second dominant coefficient [23]. The SampEn was used to capture the periodicity of the gait with a higher value indicating less periodicity [22]. 

### 2.8. Machine Learning

Machine learning can be used to evaluate the accuracy of measurement methods. This current study used Fisher’s linear discriminant analysis (LDA), artificial neural network (ANN), support vector machines (SVM), and random forest (RF). The LDA is the oldest classifier, and it is used to find a linear combination, which characterizes two or more classes of objects or events. The ANN has been used extensively in classification problems, and it can be understood as a parallel-distributed processing system. This study used multilayer perception (MLP) as it is the most used and powerful neural network [24]. The SVM is a discriminative classifier that can efficiently perform both linear and non-linear classification. The last classifier used in the current study is RF, which is one of the decision-tree’s classifiers that improves the classification performance of a single-tree classifier by combining the bootstrap aggregating method and randomization in the selection of segmenting data nodes in the construction of a decision tree [25]. The majority vote of the different decisions provided by each tree constituting the forest is used to assign the new observation vector to a class. In the comparative studies, RF outperformed the other classifiers [26]. However, RF requires large amounts of labeled data to achieve high performance.

## 3. Results

### 3.1. Evaluation Using Dynamic Stability

Dynamic stability can distinguish the different stability patterns due to ageing and illness. However, the approach cannot detect the difference between normal walking and walking with obstacles in healthy subjects, as shown in Table 2. For young adults, the significant differences were observed in fall-like motions when compared to normal walking and walking with obstacles for the ratio of RMS, step regularity, and stride regularity in all axes for the ankle data. Conversely, for the knee data, significant differences were observed in fall-like motions when compared to normal walking and walking with obstacles for the ratio of RMS, step regularity in the vertical (VT) and anteroposterior (AP) axes, and stride regularity in the vertical axis. For older adults, significant differences were observed in fall-like motions when compared to normal walking and walking with obstacles for the ratio of RMS in the VT and AP axes, step and stride regularity in all axes, and sample entropy in the VT and mediolateral (ML) axes for the ankle data. Significant differences of the knee data for older adults were only observed in fall-like motions when compared to normal walking and walking with obstacles for the ratio of RMS in the VT and AP axes and sample entropy in the VT axis. 

As shown in Table 2, the parameters of dynamic stability evidently did not distinguish the difference between normal walking and walking with obstacles. This was potentially caused by the slight difference in the stability pattern between normal walking and walking with obstacles in healthy subjects. 

### 3.2. Development of Postural Stability Index

Each resultant of the x-, y-, and z-axes of the acceleration signal was decomposed using the 8-modes EEMD. This study used the resultant as opposed to a single axis to avoid information loss. Body sway indicates poor postural stability-ability and can occur in any axis of the signal. The complexity index of each IMF was calculated and subsequently normalized by dividing the complexity index of each IMF by the total complexity index of all IMFs to minimize the influence of individual differences. 

The postural stability index (PSI) is defined as follows:

(6) $$PSI=\frac{CI\;of\;IMF3}{CI\;of\;IMF1+CI\;of\;IMF2+\ldots +CI\;of\;IMF6}$$

where *CI* denotes the complexity index obtained from the MSE.

The IMF3 was selected as the dominant IMF considering its frequency is closely related to the frequency of walking. Based on past studies, the range of walking frequency was 1.4–2.5 Hz while less stable movement exhibited a lower frequency [27,28]. Table 3 shows the frequency of each IMF component of the walking data. The frequency of IMFs was obtained from the instantaneous frequency of the Hilbert-Huang transform.

The results of the Pearson correlation showed that the IMF3 was correlated with the gait variability parameters of dynamic stability, as shown in Table 4. Step and stride variability in all axes were selected to represent gait variability. As shown in Table 4, based on the ankle data, IMF3 indicates more correlations than the other IMFs. However, the correlations between IMF3 and dynamic stability parameters were mostly negative. This implies that the IMF3 decreased with increases in gait variability. Conversely, based on the knee data, IMF1 to IMF3 were positively correlated in most dynamic stability parameters except for step and stride regularity in the vertical and anteroposterior axes. Although all three IMFs were correlated with the gait variability parameters, the correlation between dynamic stability and IMF3 exceeded those of the other IMFs.

## 4. Discussion

The differences of the SSI and PSI values for both young and older adults are shown in Table 5. The Mann Whitney test was used to compare the differences between the two groups. The significant difference between young and older adults were only observed in normal walking and walking with obstacles with the sensor location corresponding to the ankle. The results between the groups were similar, and this is explained by the fact that both groups were healthy. Unintentional falls were absent during the experiment. The Wilcoxon test was used, and significant differences were observed in all activities for the PSI (*p* = 0.005). Conversely, there was no significant difference for the SSI (*p* > 0.05).

The Wilcoxon test for the SSI on the ankle and knee did not indicate a significant difference between different activities (*p* > 0.05). Conversely, the dynamic stability did not distinguish between normal walking and walking with obstacles (Table 2). These findings are in agreement with the comparisons of the postural stability indexes shown in Table 6.

The aim of this study was to develop a measure to quantify walking stability in healthy subjects. The dynamic stability and the SSI were used as comparisons to evaluate the sensitivity of the PSI. The differences between normal walking and walking with obstacles in healthy subjects were not as evident as the differences between fallers and non-fallers. However, the PSI and step regularity in the ML axis distinguished between normal walking and walking with obstacles. 

As shown in Table 6, the PSI using EEMD outperformed the other methods using wavelets decomposition. Step regularity in the ML axis was selected to represent the dynamic stability approach because it is the only parameter that can differentiate between normal walking, walking with obstacles, and fall-like motions (Table 2a). There was no decomposition process for dynamic stability since it used the original signal data.

To determine the location of the sensor that is more sensitive, the results of the PSI for both groups of subjects were grouped based on the ankle and knee. The comparison indicated that the knee group performed better than the ankle group. Thus, the knee corresponds to a better sensor location than the ankle with an accuracy of 82.22% based on the ANN.

The original SSI (using EEMD) performed the worst in both age groups. The poor performance of the SSI happened because the SSI only considers the vertical axis data. Conversely, the PSI and dynamic stability consider the data in all axes since instability can occur in any axis. However, other than the ankle data of young adults, the accuracies for dynamic stability were generally low. The poor performance of the SSI and dynamic stability for older adults potentially occurred because those methods could not distinguish between the different activities of healthy subjects. Thus, the activities were not classified correctly.

Interestingly, the performance of the PSI dropped when using DWT and WPD as the decomposition method. Conversely, the performance of the SSI increased although it was not as good as the performance of the PSI with EEMD. Other than that, the performance of the ankle improved while the performance of the knee decreased. The poor performance of wavelet decomposition compared to EEMD might be because of the short data records (60 s). Other than that, EEMD was able to estimate the subtle changes that were obtained via the first temporal derivative of the phase angle time series, scaled by the sampling rate. The decomposition by wavelet was not optimal because of frequency smoothing and it assumed frequency stationarity during the time span of the wavelet. The results in the current study are different from a previous study evaluating the performance of EMD and wavelets [12], because the EMD in the previous study could not determine the number of IMFs in the signal and there was mode mixing issues in the EMD.

In contrast to the previous study [12], the decompositions by WPD resulted in the lowest accuracy compared to EEMD and DWT. The DWT performed better than WPD in the current study, and this may be a result of differences in the sampling frequency; 30 Hz in this study and 256 Hz in the previous study [12]. Further, the nature of gait and brain signals are different. Another study by Barralon et al. [29] showed that decomposition using a discrete wavelet was more efficient than a continuous wavelet for gait signals with a 20 Hz sampling rate.

### Walking Stability Determination

To better quantify the different stability states, the PSI values of the normal walking, walking with obstacles, and fall-like motions were evaluated. The PSI of the normal walking was used to determine the postural stability limit of each subject with the assumption that normal walking was at least 80% of the upper limit. Subsequently, the scales were developed by normalizing each PSI of the less stable movement to the upper limit value. There were small differences between young and older adults. This indicated that the MSE can eliminate the range variations between young and older adults. The normalization was calculated individually, and thus the similarity in the ranges was not equal to the same index values for all subject groups. The values of the MSE in young subjects were generally higher than those in older adults although the percentage of each movement when compared to the upper limit of postural stability for both young and older groups, were significantly similar. Therefore, the stability scales for both young and older adults can be unified as follows.

80–100%: Stable.

70–79%: Fairly stable, requires minor attention.

45–69%: Unstable, requires high attention.

<45%: Danger, may cause fall.

To determine the walking stability of the subjects, the free-walking data were evaluated using the stability index. The evaluation was divided into two parts, namely general stability determination and segmented stability state determination. In the general stability determination, the stability was determined from the data across the entire 60 s period. Conversely, in the segmented determination, stability was evaluated every 10 s.

Twenty subjects (10 young adults, 10 older adults) were asked to perform a free-walking task to determine the stability of their walk in normal circumstances. The subjects were instructed to move as they liked without any intervention. As shown in Table 6, there was one unstable movement for the ankle and five unstable movements for the knee. During the experiment, the young adults were in a fit condition and did not exhibit any problems during the free-walking performance, and they tended to walk normally in almost a straight line. However, the older adults tended to move around the circle. However, only subject E3 admitted feeling dizzy while performing the free-walking task. The results for the subject, E3, with a sensor on the knee, confirmed the real condition of the subject during the experiment.

With respect to the subject, E7, complaints related to the free-walking task were absent. However, this subject walked slower when compared to that in the normal walking and walking with obstacles tasks. This subject also paused several times during his free-walking task. This potentially occurred because the instruction involved walking freely as per the subjects’ wishes, and thus the subject, E7, was potentially not sufficiently motivated to perform his best in the task.

Other unstable movements were detected on the knee for subjects, Y3, Y5, E1, and E3. With respect to the situations during the experiment, the results did not indicate those subjects performed poorly in the free-walking task. With respect to Table 6, the knee data performed better than the ankle data. Thus, the knee is a more reliable sensor location since the accuracies on the knee exceeded those on the ankle. This is potentially the reason why the stability determination of the free-walking task indicated that more unstable movements are detected on knee. The acceleration values varied, although the same type of sensor was used to simultaneously detect similar movements on a subject. The placement of the sensor on the body of the subject significantly affected the performance of the accelerometer. The placement on the ankle was potentially less sensitive than that on the knee.

Although the stability of a movement can be considered as stable in general, it is possible that the postural stability quality of the movement is not the same all the time. Less stable movement can occur in a particular time interval. To evaluate the quality of the movement, the free-walking data of the subjects, Y3, Y5, E1, E3, and E7, were further analyzed. The sensor location on the knee was more accurate, and thus only the knee data were analyzed to determine the stability states. Thus, 60 s of walking data was divided into six segments, and postural stability was evaluated every 10 s, as shown in Table 7.

As shown in Table 8, changes occurred in the postural stability within 60 s of walking for all subjects. For example, the general stability state for the subject, Y3, was unstable. However, based on the segmented stability states, the movement was not always unstable. The first 10 s was stable and, subsequently, the stability state dropped to one in the dangerous category. Fortunately, the stability state improved to unstable then alternated back and forth between the danger and unstable category. The segmented stability state indicated that the subject, Y3, evidently attempted to maintain stability to avoid falling.

The PSI was able to evaluate the postural stability states of movement. However, given the individual differences in human movement, it was only relevant to individually evaluate the movement. The approach represented the characteristics of the stability of a particular subject. However, it was not possible to use the PSI to immediately analyze the movement. Stable state data are required to categorize the movement stability of the subject in question.

## 5. Conclusions

Quantification of human stability is required to understand the mechanism underlying balance control. The contribution of this study involves providing a novel measure of the postural stability index (PSI) to distinguish between different postural stability states in healthy individuals. The PSI discriminated between normal walking and walking with obstacles in healthy subjects while SSI and dynamic stability did not. A previous study indicated that the SSI differentiates non-fallers from fallers. However, the SSI did not capture the differences between two walking tasks in healthy subjects. This potentially occurred because the differences were excessively small, and the SSI algorithm only used vertical acceleration data in the evaluation. Conversely, the PSI used the resultant of the three-axes acceleration data by assuming that postural sway can occur in any axis.

The present study involved several limitations as follows: (1) The present study used normal walking data to determine the upper postural stability limit. Therefore, the PSI cannot be used to quantify stability without existing normal walking data; (2) the present study used six IMFs to develop the PSI, and thus future studies are required to examine the applicability of the PSI for other decompositions with a different number of IMFs; (3) the present study used wearable accelerometers, and thus future studies are necessary to investigate the effect of the sensitivity of the accelerometers on the PSI; and (4) with respect to older adults who did not perform free-falls due to safety concerns, a past study compared the intentional and unintentional falls and indicated that the difference between the fall-like motions and falling only corresponds to the inclination angles [30]. Therefore, if the evaluation does not include such angles, the fall-like motions can be used to represent the falls in general.

In conclusion, the results of the present study suggest that the EEMD and MSE algorithm can be utilized to quantify postural stability in healthy subjects. The PSI method adequately measures different postural stability states in healthy subjects.

## Figures and Tables

**Table 1:** Descriptive statistics of the subjects.

	Young Adults	Older Adults	*p*-Value
Age (years)	24 (0.88)	70 (5.27)	<0.001 *
Gender (% Female)	50%	60%	0.66
Height (cm)	164 (6.84)	160 (9.93)	0.36
Weight (kg)	59 (10.67)	60 (9.12)	0.79

* *p*-values were obtained using the Mann Whitney test. For the SSI and the new PSI, higher scores indicate better performance.

**(a):** 

Measures	Axis	NW	WO	FLM	FF	*p*-Value		
						NW-WO	NW-FLM	WO-FLM
Ankle Data								
Ratio of RMS	VT	0.50 (0.05)	0.48 (0.03)	0.64 (0.06)	0.64 (0.06)	0.16	<0.001	<0.001
	ML	0.64 (0.03)	0.62 (0.05)	0.57 (0.13)	0.57 (0.13)	0.51	0.03	0.03
	AP	0.59 (0.05)	0.62 (0.03)	0.48 (0.05)	0.48 (0.05)	0.19	0.04	0.009
Step regularity	VT	0.61 (0.09)	0.58 (0.08)	0.11 (0.06)	0.46 (0.23)	0.53	<0.001	<0.001
	ML	0.61 (0.07)	0.53 (0.09)	0.16 (0.09)	0.48 (0.11)	0.04	<0.001	<0.001
	AP	0.54 (0.10)	0.51 (0.09)	0.20 (0.11)	0.36 (0.09)	0.32	<0.001	<0.001
Stride regularity	VT	0.34 (0.11)	0.27 (0.08)	0.06 (0.05)	0.29 (0.16)	0.18	<0.001	<0.001
	ML	0.44 (0.09)	0.39 (0.07)	0.06 (0.04)	0.27 (0.13)	0.07	<0.001	<0.001
	AP	0.39 (0.10)	0.35 (0.08)	0.05 (0.03)	0.17 (0.10)	0.10	<0.001	<0.001
Sample entropy	VT	0.15 (0.11)	0.10 (0.03)	0.13 (0.06)	0.04 (0.01)	0.19	0.77	0.16
	ML	0.15 (0.13)	0.10 (0.03)	0.13 (0.07)	0.05 (0.03)	0.26	0.76	0.42
	AP	0.18 (0.27)	0.10 (0.03)	0.13 (0.06)	0.06 (0.03)	0.33	0.62	0.14
Knee Data								
Ratio of RMS	VT	0.43 (0.06)	0.45 (0.05)	0.69 (0.04)	0.55 (0.08)	0.20	<0.001	<0.001
	ML	0.66 (0.04)	0.64 (0.03)	0.56 (0.03)	0.65 (0.08)	0.25	<0.001	<0.001
	AP	0.61 (0.04)	0.46 (0.05)	0.51 (0.08)	0.52 (0.04)	0.45	<0.001	<0.001
Step regularity	VT	0.34 (0.14)	0.36 (0.14)	0.22 (0.12)	0.60 (0.12)	0.33	0.07	0.02
	ML	0.57 (0.11)	0.55 (0.10)	0.34 (0.05)	0.52 (0.14)	0.60	<0.001	<0.001
	AP	0.51 (0.10)	0.54 (0.09)	0.33 (0.10)	0.47 (0.15)	0.16	<0.001	<0.001
Stride regularity	VT	0.09 (0.06)	0.13 (0.07)	0.08 (0.04)	0.31 (0.14)	0.05	0.64	0.09
	ML	0.29 (0.11)	0.29 (0.12)	0.11 (0.05)	0.25 (0.13)	0.93	<0.001	<0.001
	AP	0.24 (0.09)	0.27 (0.08)	0.14 (0.08)	0.24 (0.11)	0.11	0.01	0.005
Sample entropy	VT	0.09 (0.05)	0.20 (0.11)	0.11 (0.05)	0.04 (0.03)	0.02	0.30	0.02
	ML	0.07 (0.03)	0.11 (0.05)	0.12 (0.04)	0.06 (0.04)	0.01	0.003	0.58
	AP	0.06 (0.02)	0.14 (0.08)	0.11 (0.04)	0.56 (0.03)	0.02	0.02	0.33

**(b):** 

Measures	Axis	NW	WO	FLM	*p*-Value		
					NW-WO	NW-FLM	WO-FLM
Ankle Data							
Ratio of RMS	VT	0.52 (0.05)	0.48 (0.05)	0.60 (0.06)	0.08	0.002	<0.001
	ML	0.56 (0.03)	0.58 (0.05)	0.56 (0.06)	0.19	0.39	0.19
	AP	0.64 (0.03)	0.65 (0.04)	0.56 (0.09)	0.30	0.007	0.005
Step regularity	VT	0.48 (0.21)	0.46 (0.22)	0.28 (0.15)	0.41	0.012	0.02
	ML	0.55 (0.14)	0.54 (0.16)	0.41 (0.09)	0.46	0.008	0.02
	AP	0.55 (0.11)	0.53 (0.16)	0.39 (0.15)	0.40	0.01	0.03
Stride regularity	VT	0.21 (0.14)	0.22 (0.15)	0.12 (0.08)	0.46	0.04	0.04
	ML	0.35 (0.17)	0.35 (0.17)	0.15 (0.11)	0.49	0.003	0.003
	AP	0.36 (0.15)	0.35 (0.14)	0.14 (0.14)	0.47	0.002	0.001
Sample entropy	VT	0.09 (0.04)	0.11 (0.04)	0.06 (0.03)	0.05	0.03	<0.001
	ML	0.08 (0.04)	0.10 (0.04)	0.06 (0.03)	0.06	0.10	0.001
	AP	0.11 (0.09)	0.12 (0.06)	0.12 (0.17)	0.38	0.48	0.45
Knee Data							
Ratio of RMS	VT	0.47 (0.06)	0.47 (0.10)	0.60 (0.08)	0.47	<0.001	0.001
	ML	0.58 (0.03)	0.61 (0.04)	0.56 (0.06)	0.08	0.12	0.02
	AP	0.66 (0.03)	0.63 (0.07)	0.56 (0.06)	0.16	<0.001	0.01
Step regularity	VT	0.38 (0.18)	0.43 (0.22)	0.34 (0.18)	0.32	0.31	0.18
	ML	0.56 (0.12)	0.61 (0.10)	0.52 (0.12)	0.13	0.25	0.04
	AP	0.55 (0.14)	0.59 (0.11)	0.53 (0.11)	0.27	0.36	0.14
Stride regularity	VT	0.14 (0.10)	0.21 (0.21)	0.15 (0.12)	0.16	0.38	0.22
	ML	0.30 (0.09)	0.36 (0.12)	0.21 (0.18)	0.14	0.08	0.02
	AP	0.29 (0.12)	0.31 (0.09)	0.30 (0.19)	0.37	0.48	0.42
Sample entropy	VT	0.13 (0.05)	0.14 (0.04)	0.08 (0.04)	0.28	0.02	0.001
	ML	0.09 (0.05)	0.08 (0.02)	0.07 (0.05)	0.24	0.24	0.40
	AP	0.09 (0.05)	0.07 (0.02)	0.08 (0.06)	0.25	0.41	0.37

VT: vertical (y-axis), ML: mediolateral (x-axis), AP: anteroposterior (z-axis), NW: normal walking, WO: walking with obstacles, FLM: fall-like motions, FF: free-falling.

**Table 3:** The average frequencies of intrinsic mode functions (IMFs) of walking data decomposed by ensemble empirical mode decomposition (EEMD).

IMF	Average Frequency
1	6.67 Hz
2	2.49 Hz
3	1.48 Hz
4	0.82 Hz
5	0.36 Hz
6	0.22 Hz

**Table 4:** Correlations between the step stability index (SSI), the postural stability index (PSI), the intrinsic mode functions (IMFs), and dynamic stability.

		IMF1	IMF2	IMF3	IMF4	IMF5	IMF6
		r (*p*-value)	r (*p*-value)	r (*p*-value)	r (*p*-value)	r (*p*-value)	r (*p*-value)
Ankle Step Reg-ML	All Subjects	NS	0.28 (<0.001)	0.29 (<0.001)	0.2 (<0.001)	0.23 (<0.001)	0.28 (<0.001)
	Young Adults	NS	−0.26 (<0.001)	−0.21 (<0.001)	−0.24 (<0.001)	NS	NS
	Older Adults	NS	−0.21 (<0.001)	−0.42 (<0.001)	NS	−0.24 (<0.001)	NS
Step Reg-VT	All Subjects	NS	0.21 (<0.001)	NS	NS	NS	NS
	Young Adults	NS	NS	NS	−0.23 (<0.001)	NS	−0.22 (<0.001)
	Older Adults	NS	−0.29 (<0.001)	−0.25 (<0.001)	NS	NS	NS
Step Reg-AP	All Subjects	NS	NS	0.22 (<0.001)	NS	NS	NS
	Young Adults	NS	NS	NS	NS	NS	−0.22 (<0.001)
	Older Adults	NS	NS	−0.37 (<0.001)	NS	−0.28 (<0.001)	NS
Stride Reg-ML	All Subjects	NS	NS	0.27 (<0.001)	NS	NS	NS
	Young Adults	NS	NS	−0.22 (<0.001)	NS	NS	NS
	Older Adults	−0.20 (<0.001)	NS	−0.46 (<0.001)	NS	−0.28 (<0.001)	NS
Stride Reg-VT	All Subjects	NS	0.22 (<0.001)	0.24 (<0.001)	NS	NS	NS
	Young Adults	NS	−0.21 (<0.001)	−0.24 (<0.001)	−0.27 (<0.001)	NS	−0.24 (<0.001)
	Older Adults		−0.36 (<0.001)	−0.28 (<0.001)	NS	NS	NS
Stride Reg-AP	All Subjects	NS	NS	0.25 (<0.001)	NS	NS	NS
	Young Adults	NS	NS	NS	−0.21 (<0.001)	NS	−0.22 (<0.001)
	Older Adults	NS	NS	−0.41 (<0.001)	NS	−0.34 (<0.001)	NS
Knee Step Reg-ML	All Subjects	0.45 (<0.001)	0.36 (<0.001)	0.48 (<0.001)	NS	NS	NS
	Young Adults	0.40 (<0.001)	0.45 (<0.001)	0.41 (<0.001)	0.28 (<0.001)	0.34 (<0.001)	0.33 (<0.001)
	Older Adults	0.39 (<0.001)	0.24 (<0.001)	0.57 (<0.001)	NS	NS	−0.25 (<0.001)
Step Reg-VT	All Subjects	0.24 (<0.001)	0.26 (<0.001)	0.26 (<0.001)	NS	NS	NS
	Young Adults	NS	0.33 (<0.001)	NS	NS	0.26 (<0.001)	NS
	Older Adults	0.20 (<0.001)	0.21 (<0.001)	0.49 (<0.001)	NS	NS	NS
Step Reg-AP	All Subjects	0.38 (<0.001)	0.30 (<0.001)	0.42 (<0.001)	NS	NS	NS
	Young Adults	NS	0.35 (<0.001)	0.44 (<0.001)	0.39 (<0.001)	0.27 (<0.001)	0.20 (<0.001)
	Older Adults	0.43 (<0.001)	0.26 (<0.001)	0.29 (<0.001)	NS	NS	−0.46 (<0.001)
Stride Reg-ML	All Subjects	0.36 (<0.001)	0.28 (<0.001)	0.35 (<0.001)	NS	NS	NS
	Young Adults	0.39 (<0.001)	0.37 (<0.001)	0.29 (<0.001)	0.24 (<0.001)	0.29 (<0.001)	0.25 (<0.001)
	Older Adults	0.23 (<0.001)	0.20 (<0.001)	0.43 (<0.001)	NS	NS	NS
Stride Reg-VT	All Subjects	NS	NS	NS	NS	NS	NS
	Young Adults	NS	NS	NS	NS	NS	−0.20 (<0.001)
	Older Adults	NS	NS	0.32 (<0.001)	NS	0.36 (<0.001)	NS
Stride Reg-AP	All Subjects	0.27 (<0.001)	NS	0.25 (<0.001)	NS	NS	NS
	Young Adults	NS	0.26	0.27	0.25	NS	NS
	Older Adults	0.26 (<0.001)	NS	NS	NS	NS	−0.23 (<0.001)

Reg = regularity, NS = not significant.

**Table 5:** Group differences of the SSI and the new PSI measure. Data are reported as mean (SD).

	Young Adults	Older Adults	*p*-Value
Ankle Data			
SSI Normal walking	0.13 (0.02)	0.12 (0.03)	0.50
SSI Walking + obstacles	0.13 (0.03)	0.12 (0.02)	0.33
SSI Fall-like motions	0.11 (0.02)	0.11 (0.02)	0.50
SSI Free-fall	0.29 (0.06)	N/A	N/A
PSI Normal walking	0.33 (0.04)	0.25 (0.04)	<0.001
PSI Walking + obstacles	0.27 (0.04)	0.19 (0.03)	0.001
PSI Fall-like motions	0.16 (0.03)	0.14 (0.02)	0.16
PSI Free-fall	0.12 (0.03)	N/A	N/A
Knee Data			
SSI Normal walking	0.09 (0.01)	0.10 (0.02)	0.14
SSI Walking + obstacles	0.09 (0.01)	0.10 (0.02)	0.13
SSI Fall-like motions	0.12 (0.02)	0.11 (0.03)	0.60
SSI Free-fall	0.28 (0.04)	N/A	N/A
PSI Normal walking	0.28 (0.06)	0.27 (0.03)	0.60
PSI Walking + obstacles	0.21 (0.06)	0.19 (0.02)	0.41
PSI Fall-like motions	0.14 (0.04)	0.14 (0.02)	0.51
PSI Free-fall	0.10 (0.04)	N/A	N/A

**Table 6:** Accuracy for activity recognition on the ankle and knee.

	LDA			ANN			SVM			RF		
	DS	SSI	PSI	DS	SSI	PSI	DS	SSI	PSI	DS	SSI	PSI
Ensemble Empirical Mode Decomposition (EEMD)												
Young—Ankle	86.67	66.67	86.67	82.22	60.00	88.89	82.22	57.78	82.22	73.33	53.33	82.22
Young—Knee	68.89	64.44	68.89	68.89	73.33	72.22	68.89	64.44	64.44	74.44	71.11	68.89
Elder—Ankle	66.67	51.11	77.78	60.00	51.11	80.00	60.00	42.22	71.11	48.89	46.67	75.56
Elder—Knee	57.78	53.33	93.33	64.44	53.33	93.33	60.00	53.33	71.11	53.33	42.22	**93.33**
All—Ankle	71.11	54.44	74.44	71.11	53.33	80.00	68.89	57.78	74.44	62.22	51.11	74.44
All—Knee	63.33	60.00	82.22	63.33	60.00	82.22	58.89	62.22	74.44	61.11	50.00	81.11
Discrete Wavelet Transform (DWT)												
Young—Ankle	86.67	80.00	68.89	82.22	80.00	70.00	82.22	71.11	66.67	73.33	75.56	57.78
Young—Knee	68.89	55.56	44.44	68.89	60.00	55.56	68.89	55.56	48.89	74.44	55.56	66.67
Elder—Ankle	66.67	71.11	75.56	60.00	71.11	68.89	60.00	55.56	68.89	48.89	55.56	71.11
Elder—Knee	57.78	62.22	71.11	64.44	57.78	68.89	60.00	51.11	68.89	53.33	44.44	64.44
All—Ankle	71.11	76.67	71.11	71.11	75.56	71.11	68.89	66.67	71.11	62.22	66.67	63.33
All—Knee	63.33	57.78	51.11	63.33	57.78	56.67	58.89	57.78	51.11	61.11	48.89	63.33
Wavelet Packet Decomposition (WPD)												
Young—Ankle	86.67	70.00	56.67	82.22	73.33	61.11	82.22	71.11	60.00	73.33	64.44	60.00
Young—Knee	68.89	46.67	46.67	68.89	48.89	53.33	68.89	60.00	48.89	74.44	51.11	51.11
Elder—Ankle	66.67	60.00	50.00	60.00	55.56	51.11	60.00	60.00	46.67	48.89	51.11	60.00
Elder—Knee	57.78	53.33	51.11	64.44	57.78	53.33	60.00	57.78	55.56	53.33	53.33	55.56
All—Ankle	71.11	63.33	63.33	71.11	61.11	63.33	68.89	63.33	55.56	62.22	57.78	65.56
All—Knee	63.33	57.78	44.44	63.33	58.89	48.89	58.89	55.56	50.00	61.11	56.67	52.22

LDA = Fisher’s linear discriminant analysis, ANN = artificial neural network, SVM = support vector machines, RF = random forest, DS = dynamic stability, All = all subjects.

**Table 7:** General walking stability determination.

Subject	Upper Limit		Free Walking		Normalization		Category	
	Ankle	Knee	Ankle	Knee	Ankle	Knee	Ankle	Knee
Y1	0.44	0.33	0.39	0.28	88.46%	84.19%	S	S
Y2	0.33	0.39	0.25	0.30	76.39%	77.44%	F	F
Y3	0.35	0.49	0.26	0.25	73.76%	51.41%	F	U
Y4	0.41	0.40	0.34	0.35	83.16%	87.98%	S	S
Y5	0.46	0.35	0.34	0.24	73.50%	67.00%	F	U
Y6	0.40	0.27	0.35	0.23	88.50%	85.11%	S	S
Y7	0.45	0.28	0.35	0.24	78.66%	85.00%	F	S
Y8	0.39	0.38	0.31	0.32	80.78%	83.99%	S	S
Y9	0.44	0.22	0.35	0.21	79.05%	93.97%	F	S
Y10	0.44	0.32	0.37	0.26	84.07%	81.90%	S	S
E1	0.36	0.32	0.31	0.21	85.92%	65.40%	S	U
E2	0.35	0.30	0.29	0.27	82.42%	89.88%	S	S
E3	0.24	0.35	0.17	0.18	70.93%	51.53%	F	U
E4	0.28	0.43	0.24	0.34	84.16%	80.47%	S	S
E5	0.27	0.33	0.24	0.29	90.19%	89.20%	S	S
E6	0.26	0.37	0.23	0.29	88.71%	76.25%	S	F
E7	0.38	0.31	0.16	0.12	41.20%	39.34%	D	D
E8	0.29	0.30	0.25	0.25	85.35%	82.50%	S	S
E9	0.32	0.29	0.26	0.22	79.38%	77.60%	F	F
E10	0.32	0.33	0.28	0.27	87.47%	83.05%	S	S

Category = stability category, S = stable, F = fairly stable, U = unstable, D = danger, Y = young adults, E = older adults.

**Table 8:** Segmented walking stability determination.

Time Interval	Y3		Y5		E1		E3		E7	
	Norm	Cat	Norm	Cat	Norm	Cat	Norm	Cat	Norm	Cat
0–10s	84.92%	S	94.64%	S	85.21%	S	12.00%	D	55.50%	U
10–20 s	36.78%	D	56.15%	U	83.43%	S	44.00%	D	44.22%	D
20–30 s	46.30%	U	90.36%	S	85.90%	S	37.35%	D	54.63%	U
30–40 s	36.08%	D	71.90%	F	36.28%	D	80.34%	S	11.25%	D
40–50 s	66.52%	U	70.51%	F	78.99%	F	33.22%	D	13.77%	D
50–60 s	42.01%	D	44.28%	D	46.21%	U	68.95%	U	23.84%	D

Norm = normalization, Cat = stability category, S = stable, F = fairly stable, U = unstable, D = danger, Y3 = young subject#3, Y5 = young subject#5, E1 = older adult#1, E3 = older adult#3, E7 = older adult#7.
